# Aplitic Granite Waste as Raw Material for the Production of Outdoor Ceramic Floor Tiles

**DOI:** 10.3390/ma15093145

**Published:** 2022-04-26

**Authors:** Milica Vidak Vasić, Nevenka Mijatović, Zagorka Radojević

**Affiliations:** Institute for Testing of Materials IMS, Bulevar Vojvode Mišića 43, 11000 Belgrade, Serbia; nevenka.mijatovic@institutims.rs (N.M.); zagorka.radojevic@institutims.rs (Z.R.)

**Keywords:** ceramic tile, aplitic granite waste, flux, recycling

## Abstract

One of the significant problems in the production of ceramic tiles is the very high consumption of natural resources such as clay, feldspar, and quartz. The possibility of replacing part of the formulation of ceramic batches is of great importance. In this research, the possibility of using aplitic granite waste from dimensional stone production was analyzed in detail. The waste is considered a low-cost substitute for feldspar in Serbia. The milled powdery waste was analytically tested to reveal its chemical and mineralogical contents, particle size distribution, and other important properties. The ceramic tiles containing aplitic granite waste (GW) and GW/raw clay mixture (CGW) were hydraulically pressed, and the ceramic and technological properties determined. This waste can act as a filler while forming, drying, and firing, since the high content of quartz helps to control the shrinkage and acts as a fluxing agent in high temperatures due to its feldspathic nature. The waste was found favorable in the production of ceramic tiles, as the gained values of modulus of rupture and water absorption were 28.68 MPa and 1.33%, respectively. The parameters defined in the series of standards EN ISO 10545 were tested on a semi-industrial probe, determining that this combination of materials (without the addition of quartz) may be efficiently used to produce ceramic floor tiles. The usage of what would otherwise be waste material contributes to sustainable management and environmentally friendly solutions by avoiding landfilling, while at the same time it enabling the conservation of scarce natural feldspar deposits.

## 1. Introduction

Among raw materials, non-metallic minerals are consumed in a significant quantity of 52 mass% [1]. The traditional ceramic industry is one of the heaviest consumers of raw natural resources. Given that the need for tiles is constantly growing, so too is the consumption of raw materials [2,3]. Increasing concern about a secure and affordable supply of raw materials for the ceramic industry, including fluxes, is present worldwide [4]. On the other hand, the construction and mining industries are responsible for generating 36% and 25% of waste, respectively [5]. Additionally, cutting natural granite can involve there being more than 50% leftover. If used, these leftovers are, if used, mainly spent as aggregates [5,6] or in geotechnical applications [7]. Such construction applications have recently been gaining more interest [8].

Ceramic batch formulations usually include large quantities of feldspars (25–35 mass%) and quartz (15–25 mass%), the rest being clay (30–50 mass%) [9,10,11,12]. The formulations are related to the final application of tiles, which is primarily dependent on their water absorption and modulus of rupture. The floor tiles can be formed of batches containing 20–40 mass% of feldspars, 5–30 mass% of quartz, and 30–55 mass% of clay [13]. Of all the applications in ceramic and glass, most feldspar is spent on the production of ceramic tiles. Granitic rocks usually present the main source of fluxes in ceramic tile production. The consumption is estimated to be around 3.6 million tons per year from about 50 mines, which represents approximately one-fifth of the global production of fluxes [3]. The main advantages of the exploitation of granites are the large extension of deposits, which usually exhibit mild compositional variations, and a limited amount of femic minerals (mainly micas), especially in the leucocratic terms sought-after by the ceramic industry [14].

Having in mind the ongoing necessity for more environmentally friendly solutions, many researchers have dealt with incorporating waste materials to produce different kinds of ceramic tiles. There is limited data in the literature concerning the usage of granitic waste in ceramics, while an aplitic granite seems to have never been studied before. Up to 70 mass% of granite sludge is incorporated in red extruded wall ceramic tile production, thus lowering the mechanical strength [15]. Besides, a 47.5 mass% of the rock cutting waste is added to gain harder products by using a fast-firing cycle [16]. Up to 30 mass% compositions were approved for wall tile production [17]. Lately, this kind of waste, in addition to 12 mass%, is gaining more interest as a valuable raw material source in the production of traditional ceramic [18]. Cut and polished granitic waste is introduced in a quantity of up to 25 mass% in combination with eggshell (up to 15 mass%) to produce ceramic bricks [19], or in a quantity of 30 mass% in the roof tile industry [20]. Additionally, cutting and polishing granitic waste is successfully utilized to produce fly ash interlocking bricks [6]. Among other materials of a similar mineralogy, the studies presented the usage of raw natural granite [21,22], feldspathic sand [23], and the waste derived from the mining of feldspar [24]. Some studies aim to replace the natural feldspar used in batches with different materials such as wollastonite [10]. A high quantity of silica stone has been added in a quantity of up to 20% to ceramic floor tile mixtures [2]. However, most of the studies conducted only laboratory-level probes in traditional ceramics production, but not in the production of outdoor ceramic floor tiles. Besides, the scaling up to industrial studies and eventual usage is also lacking.

Particularly important is the possibility of using granitic waste from the extraction of ornamental dimension stones. This path can be a triple win: providing adequate fluxes for ceramic tiles, preserving the feldspar deposits, and, at the same time, removing piles of waste with environmental and economic benefits [25]. In this framework, the situation in the Balkan countries is worth noting, since the available resources of ceramic fluxes are restricted to small pegmatite and albitite deposits, many of which are no longer in operation [26]. This is a limiting factor for the development of a domestic industry not heavily dependent on the import of raw materials. In a small country such as Serbia, there are several dozen quarries, both active and abandoned. The quarries in operation extract granitic rocks, which can be the source of ceramic raw materials, as ornamental and building stones. There is no data in the literature on the annual production of granite, and these data are needed to estimate the amount of aplitic waste. Additionally, there are no data on the use of this waste.

Aplitic granite waste is the form of scattered rock of low mechanical strength found in the quarry in Serbia. The main constituents are feldspars (mostly albite) and quartz, with minor amounts of micas. This waste has a filler effect because it reduces shrinkage in drying and firing due to the high content of quartz, but also as a fluxing ingredient during firing due to the dominant share of feldspars. A detailed analysis of the raw materials and technological aspects of producing the ceramic tiles is shown in this study. An adequate mixture of the aplitic waste and raw clay has been proved (by tests meeting European standards) to have good potential for reducing the consumption of natural resources, decreasing the quantity of quarry industrial waste, minimizing the raw material costs, and obtaining a good quality outdoor ceramic floor tile.

## 2. Materials and Methods

**Figure 1:**
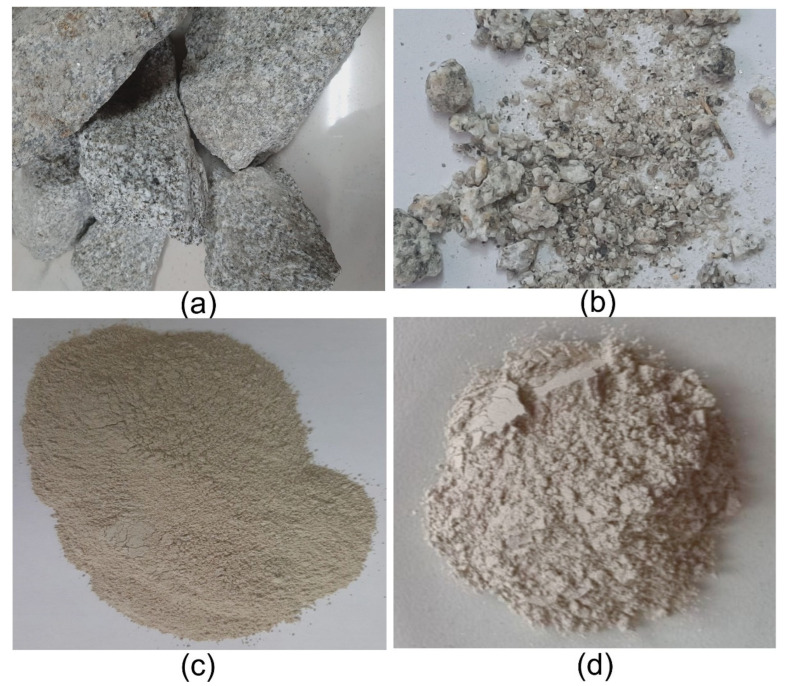
The appearance of the samples: (**a**) aplitic granite waste received as blocks (5–7 cm), (**b**) aplitic granite waste received as fragments (5–25 mm), (**c**) aplitic granite waste fraction < 0.5 mm, and (**d**) aplitic granite waste (40 mass%) and raw clay (60%) fraction < 0.5 mm.

The aplitic granite waste (GW) was from a quarry opened in the Bukulja mountain, Serbia. The GW was taken from tailings and discarded because it did not meet the mechanical quality of granite suitable for use as a natural stone for building applications. The sample of granitic material was in the form of a scattered grusified rock, in pieces of different sizes that are easily crushed by hand (Figure 1a,b). The general pattern was gray. The white and pale gray grains within the sample mass ranged up to 5 mm in size and were evenly distributed with rare minor accumulations. The black grains were up to about 1.5 mm in size. Yellow-orange and pearl-like grains were also present.

To determine the possibility of using the aplitic granite waste in the production of ceramic floor tiles, a mixture of the sample with raw ceramic clay was examined. A raw ceramic clay from Šabac (Serbia) was mixed with 40 mass% of the waste [27] to obtain a composite (CGW). The grinding of the as-received samples was done manually using a crusher. Afterward, the granulometry analysis was completed according to the standardized procedure by the combined method of hydrometry and wet sieve analysis [28]. After grinding and selecting the fraction below 0.5 mm for further use, a residue on a sieve of +63 microns in size was determined by wet sieving as a fast method of determining the fineness of the materials.

The crushed samples were dried to a constant mass and—with the help of a laboratory planetary mill—further comminuted to a granulation below 0.5 mm (Figure 1c,d). These fractions were used for further testing.

The residues on the 0.063 mm sieve were determined by a wet method and examined using a petrological polarizing microscope (Ernst Leitz, Wetzlar, Germany). The total content of carbonates (CCC) was determined by Scheibler’s volumetric method [29]. The chemical (energy dispersive X-ray fluorescence (XRF)) and mineralogical (X-ray diffraction analysis (XRD)) compositions were determined using the SpectroXepos instrument (Kleve, Germany) and Philips 1050 X-ray powder diffractometer (Amsterdam, The Netherlands), respectively, in the same way as previously described [28]. The semi-quantitative determination of minerals detected by XRD was determined by the reference intensity ratio method [30]. During the analysis in the mid-infrared part of the spectra, the FT-IR spectrophotometer Nicolet iS10 (Thermo Fisher Scientific, Waltham, MA, USA) equipped with an attenuated total reflectance accessory was applied. Details on the experimental setup are listed in the literature [31].

Behavior during the firing of the samples was examined instrumentally by dilatometry analysis (Setaram, Lyon, France) [28] and differential scanning calorimetry and thermal gravimetry (SDT Q600, TA Instruments, Hüllhorst, Germany) in the oxidizing environment [32].

About 4 mass% of moisture was added to the comminuted powders of the GW and CGW and left to homogenize for 24 h in sealed nylon bags. Immediately before molding, the masses were pressed through a 2 mm sieve to ensure the even filling of the mold. The shaping of the test bodies was performed using an Alfred Amsler hydraulic press at a pressure of 400 kg/cm^2^. The samples were wet-pressed to tiles (25 × 120 mm^2^ and 50 × 120 mm^2^). The molds were designed by the furrows to mark the place where the measuring of dimensions is to be conducted [28].

Plasticity and drying sensitivity were determined by the methods following Pfefferkorn and Bigot, respectively [29].

Drying of the formed samples was performed to a constant weight in a laboratory chamber dryer at 100 ± 5 °C. The samples were subsequently fired in a laboratory electric chamber oven in an oxidizing environment [28] at 1100, 1200, and 1250 °C. The retention time at the final temperature was 1 h.

The ceramic and technological parameters were determined in the same way as described in our previous study [28]. The number of samples of all shapes and firing temperatures was 5. Firing shrinkage was the average calculated from width and length. Water absorption and modulus of rupture were determined as suggested by the standards SRPS EN ISO 10545-3 [33] and SRPS EN ISO 10545-4 [34]. The temperatures of clinkering and sintering were determined as described in the previous study [28].

Refractoriness was determined using a standard-defined procedure as a temperature of softening of materials [35].

A ColorLite spectrophotometer instrument (model SPH870) using the high-powered LED-light source and the certified white standard was employed to determine the corresponding L*a*b* coordinates.

The micromorphologies of the dried and Au-Pd coated GW and CGW samples were examined in a high vacuum after firing at 1250 °C. A JEOL JSM 5800 scanning electron microscope equipped with energy dispersive spectroscopy (SEM-EDS) was used. The composition is determined by an Oxford Link Isis 300 with a SiLi X-ray detector calibrated using a Ni plate.

The laboratory samples serving as the technological probe (50 × 120 mm^2^) were subjected to measuring dimensions, determination of surface quality, water absorption (Isovacuum, Gabielli, Florence, Italy), and modulus of rupture (CROMETRO CR4/E1, Gabielli, Florence, Italy). Additionally, the linear thermal expansion coefficient (Dilatometro D–103, Gabielli, Italy), deep abrasion testing (Abrasimetro—CAP, Gabrielli, Florence, Italy), resistance to freeze/thaw cycles (freeze/thaw chamber, Ves elektro, Belgrade, Serbia), chemical resistance, and Pb and Cd given off by tiles were also observed (series of standards SRPS EN ISO 10545). The lead and cadmium given off by tiles were measured from the leachate by using an inductively coupled plasma (ICP) Spectro Genesis instrument equipped with Smart Analyzer Vision software. A generator of 27.12 MHz and a power of 1700 KW allowed the radial distribution of plasma with a wavelength range of 175–775 nm. High purity argon was used in all phases of the experiment (plasma initiation, as a carrier gas, and in the cooling system).

## 3. Results and Discussion

### 3.1. Characterization of Initial Materials

**Figure 2:**
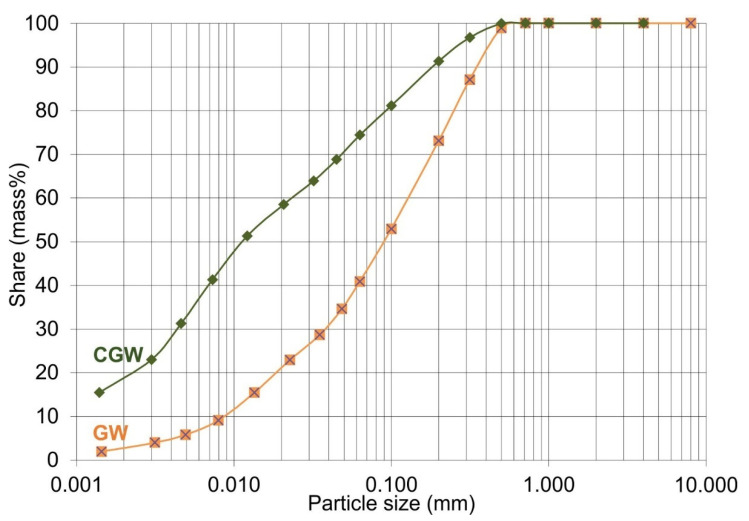
Particle size analysis of the aplitic granite waste and the composite.

The particle distribution was determined for the ground sample of disintegrated granitic waste and its mixture with the raw clay (Figure 2, Table 1).

Particle size analysis revealed that the GW belonged to sandy loam and the CGW to silt loam, both of a grey pattern. The GW showed coarser particle size distribution than the composite sample (CGW). Microscopic identification of both 0.063 mm gray-patterned sieve residues determined the powdery materials are built of quartz, feldspar, and cobwebs of brown biotite and pearl-like muscovite. Aggregate grains of a dark reddish color, most likely of iron-siltstone composition, were also present to a low extent. The samples showed no reaction in contact with 5 vol% HCl. The observed similarities between the residues are one of the factors that confirm the possibility of using both materials in the production of ceramic tiles. Both the samples contained 0.00% of total carbonates, as determined by the volumetric method.

The chemical content (Table 1) revealed that the GW mainly consisted of SiO_2_, with significant quantities of Na_2_O and K_2_O, and relatively low content of Al_2_O_3_. The increased content of fluxes makes this material very favorable in terms of obtaining high-strength ceramic tiles. The quantity of Fe_2_O_3_, TiO_2,_ and MnO seemed satisfactory to obtain a pale color [22]. The sample GW exhibited a typical chemical composition of a granite (Table 1) that is very close to the average chemical analysis of raw granite deposits from which fluxes used by the ceramic tile industry are usually recovered (Na_2_O in the range of 0.39–9.12 mass% and K_2_O in the range of 0.51–7.63 mass%) [3]. Based on the chemical composition, GW can be classified as a quartz feldspathic flux of type NKQ1, and is considered a raw granitoid suitable for application in the production of ceramic tiles [3,6]. The chemical composition of GW falls within the range of raw granites, but its iron amount is not so low as to be comparable with leucogranites [3].

The chemical content of some of the trace elements expected to leach from the materials was determined by using ICP (Table 2). We show that cadmium, chromium, copper, zinc, nickel, and arsenic are contained in a quantity lower than in some natural rocks, which would not cause problematic leaching [36]. However, the quantities detected in the bulk materials were lower than those leached out from fly ash [37]. The quantity of lead and barium might be problematic, and also possibly that of chromium. The leaching tests were done according to the procedure proposed in SRPS EN ISO 10545-15 [38], and none of these trace elements were detected in the distilled water solution.

**Figure 3:**
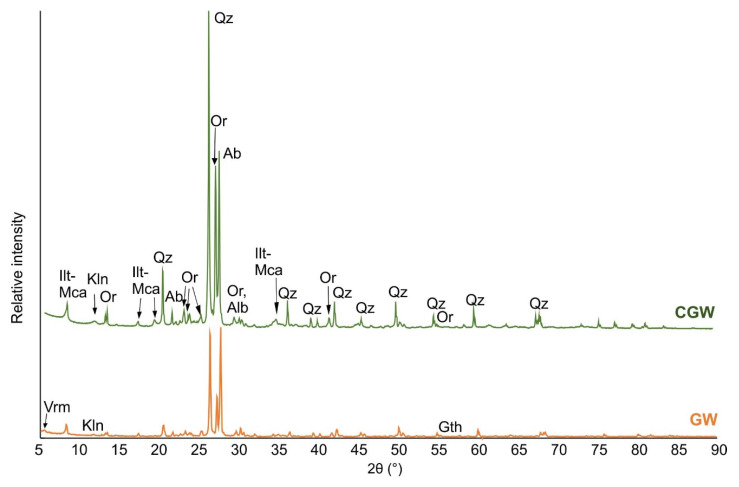
XRD pattern of the aplitic granite waste (GW) and the composite (CGW) (Ilt-Mca, illite-mica; Kln, kaolinite; Or, orthoclase; Qz, quartz; Ab, albite; Vrm, vermiculite; Gth, goethite).

Mineralogical analysis of the aplitic granite waste showed that the most common minerals were feldspars (albite and orthoclase), quartz, and illite-mica (Figure 3). Albite was dominant over other minerals, which is usual in hydrothermally altered granitoids [24,39]. The kaolinite content was low (about 2%), which indicated a low degree of kaolinization of the granite rock, and consequently a relatively young geologic age of the sediment. Additionally, dolomite, goethite, and vermiculite were detected in minor quantities (Figure 3, Table 3). For a more improved ceramic flux, it would be beneficial, although not necessary, to lower the amounts of micas and iron oxide by means such as high-intensity magnetic separation. When 60 mass% of the raw ceramic clay is introduced to 40 mass% of the GW, a dominantly quarzitic sample is obtained, containing 27 mas% of feldspars and 24 mass% of clay minerals (Figure 3, Table 3).

**Figure 4:**
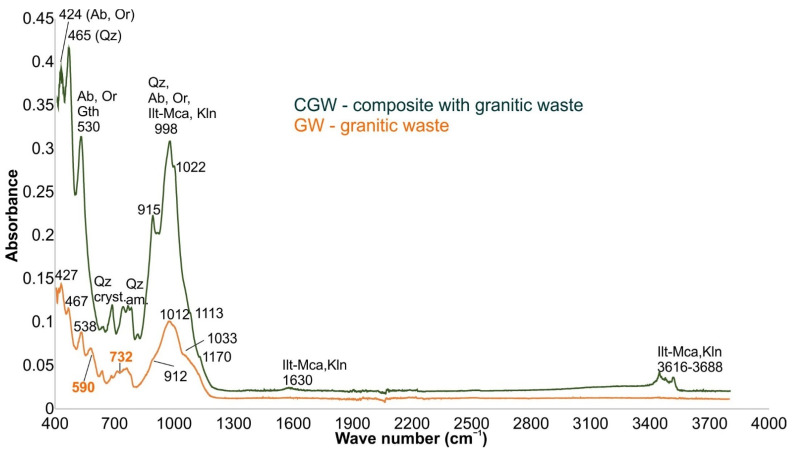
FT-IR bands of the tested samples (Ilt-Mca, illite-mica; Kln, kaolinite; Or, orthoclase; Qz, quartz; Ab, albite; Gth, goethite).

The obtained FT-IR bands in the GW and CGW samples (Figure 4) complemented the composition of the material determined by the XRD and XRF analyses. The sharp bands at about 3616, 3652, and 3688 cm^−1^ in the CGW correspond to the -OH groups’ stretching vibrations of illite-mica and some kaolinite [31]. Additionally, the mild and wide band at 1630 cm^−1^ indicates the bending vibration of the physically adsorbed water molecules to illite-mica. A weak band of illite-mica was observed at around 848 cm^−1^ [31]. All the previously mentioned peaks are missing in the GW samples.

The largest bands at 998/1012 cm^−1^ and mild shoulders at 1022/1033 cm^−1^ in the CGW and GW correspond to the Si-O asymmetrical stretching vibrations of quartz, feldspars, and clay minerals [40,41]. The prominent and mild absorption shoulders at about 915/912 cm^−1^ represent the Al-OH vibrations of the clay minerals in the CGW and GW, respectively [31]. The small bands that appeared as the shoulders near the bottom of the most intensive band (CGW:1113 and 1170 cm^−1^, GW: 1078 and 1175 cm^−1^) may be attributed to the characteristic splitting of feldspar bands [42].

The distinctive bands at 424/427 and 530/538 cm^−1^ (CGW/GW) again present the part of the footprint of feldspars [40,42]. Nonetheless, the second band at 530/538 cm^−1^ may also correspond to trace amounts of hematite [31]. Besides, in the sample GW, the band is detected at 590 cm^−1^, showing O-Si(Al)-O bending vibrations in feldspars. The same vibrations are noticed at about 652/648 cm^−1^ in both samples (CGW/GW), corresponding to orthoclase [42].

The crystalline form of quartz and symmetrical bending vibrations of the Si-O bond [31,40] is detected at 697/701 cm^−1^ in CGW/GW, being more prominent in CGW. The amorphous portion of quartz is seen as stretching vibrations in the CGW as a doublet at around 785 and 799 cm^−1^. The first band in a triplet, to which the quartz doublet builds, is detected at 760 cm^−1^ in CGW, and corresponds to feldspars [40]. Amorphous quartz is not detected in the GW sample. Several other bands corresponding to Si-O-Si deformation of quartz occur in the 465/467 cm^−1^ (CGW/GW) [31,40,41].

**Figure 5:**
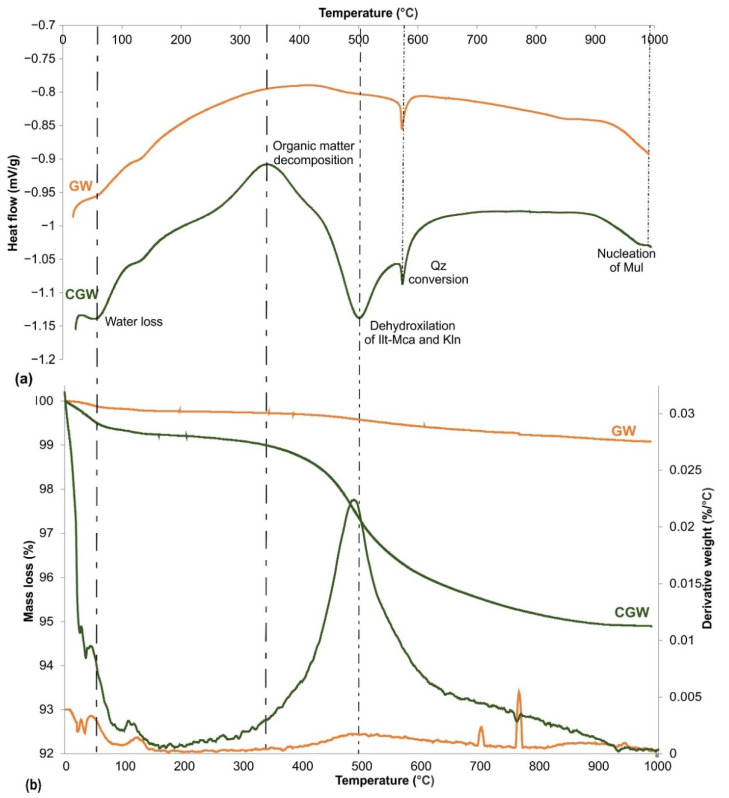
(**a**) DTA and (**b**) TGA/DTG analyses of the aplitic granite waste (GW) and the composite (CGW).

The DSC/TGA/DTG analysis of the aplitic granite waste is rarely presented in the literature. The peaks on the DSC diagram of the GW confirmed the significant presence of quartz (Figure 5a). TG analysis showed that this sample lost a small amount of water. A small mass loss, when heated to 1000 °C (below 1%), confirmed the very low content of clay mineral components. The DSC/TGA result obtained was very similar to the one published in the case of granitic rock [22]. Thus, this waste material would act as a filler in a mixture with clay by lowering the plasticity due to its high content of quartz, and as a flux at elevated temperatures due to its feldspathic nature [12,13,43,44]. Quartz is expected to increase the mechanical strength of the products by filling the porosity with melt [22]. On the other hand, the CGW sample showed a mass loss of 5.1%, which was mainly caused by the clay minerals introduced with raw clay. The removal of free water and interlayer hydroxyl groups appeared at 50 °C in the CGW (Figure 5b) and contributed to about 0.42 mass% of loss at the corresponding TGA curve. A similar effect is seen in the curve of GW but to a significantly lower extent. The combustion of a small amount of organic matter initiated at about 200 °C, gaining the exothermic maximum at 342 °C in the CGW.

The most intensive endothermic peak seen at about 500 °C corresponds to the dehydroxylation of illite-mica and some kaolinite [31] when the most intensive mass loss was also observed (Figure 5b). The ά-β structural conversion of quartz is detected at 573 °C in both the samples, being more prominent in the CGW. The sharp peak in the DTG curve at 706 °C in the GW indicates a small amount of calcite, and less intensive peaks at about 768 °C for both the samples show the presence of a minor MgCO_3_ [31]. A period of almost no thermal changes in both samples was experienced from about 600 to 889 °C, after which mild endothermic reactions occurred, presenting further structural reorganization within the materials and complete crystal water removal [31]. A low-intensity exothermic peak occurred at 983 °C in the CGW, corresponding to a small amount of the primary mullite formation.

**Figure 6:**
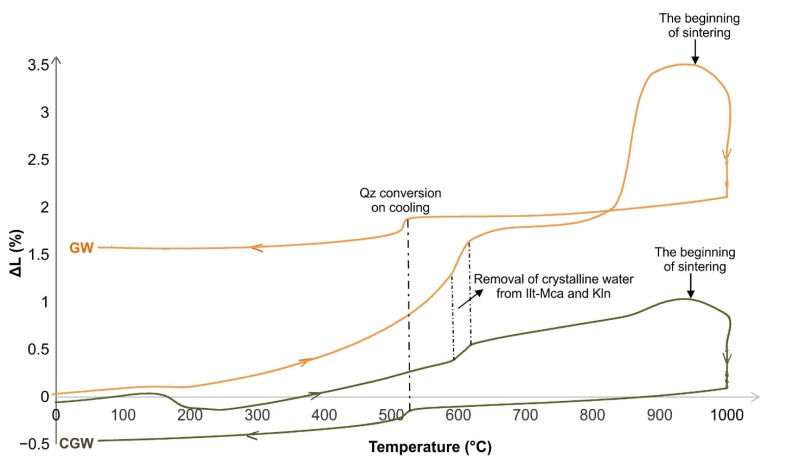
Thermo-dilatometric analyses of the aplitic granite waste (GW) and the composite (CGW).

The first known dilatometric analysis of this kind of waste is shown in the following sections. During the dilatometry testing, the GW sample was found to intensively constantly expand by 3.45% when heated to 958 °C, which was followed by fast collection, i.e., sintering (Figure 6). The effect is taken to reflect a high content of fine-grained feldspars in the material [44]. On the other side, the CGW has gently expanded by 1.08% up until 949 °C, experiencing low firing shrinkage which is very convenient for the production of ceramic tiles.

Both samples showed a mild spreading of about 0.07%; this is related to the removal of adsorbed water while firing up until about 161 °C. A period of the absence of the dimensional changes lasted up to 243 and 190 °C in the GW and CGW, respectively. A sudden shrinkage of 0.13% was observed in the CGW composite up to 228 °C. This was followed by a period of accelerating expansion to 635 °C in the GW (1.53%), and a constant and intensive expansion of a total of 0.64% in the case of CGW to 614 °C. The expansion is a consequence of the removal of crystalline water from illite-mica and kaolinite, the inversion of quartz, and the decomposition of the organic matter [31]. The typical acceleration of spreading, recorded between 614 and 635 °C in the composite sample, is associated with the final removal of the OH-groups from clay minerals [31]. After this period, both samples expanded slightly, i.e., by 0.32% to 836 °C in the case of GW and by 0.17% to 930 °C in the case of CGW. The sample GW experienced a notable expansion of 1.45% in the region between 836 and 896 °C, which was followed by an interval without major changes lasting up to 958 °C. Final shrinkage began at 958 and 949 °C in the GW and CGW, respectively. The later shrinkage was caused by the γ-alumina and mullite formation in the CGW [3]. The more pronounced shrinkage of 1.03% at the final temperature in the case of the GW sample was a consequence of the sintering of feldspars, which begins at 900 °C [3]. While cooling, both of the samples shrank by about 0.5% due to the relatively high quantity of quartz [31].

### 3.2. The Behavior in Shaping, Drying and Firing

**Figure 7:**
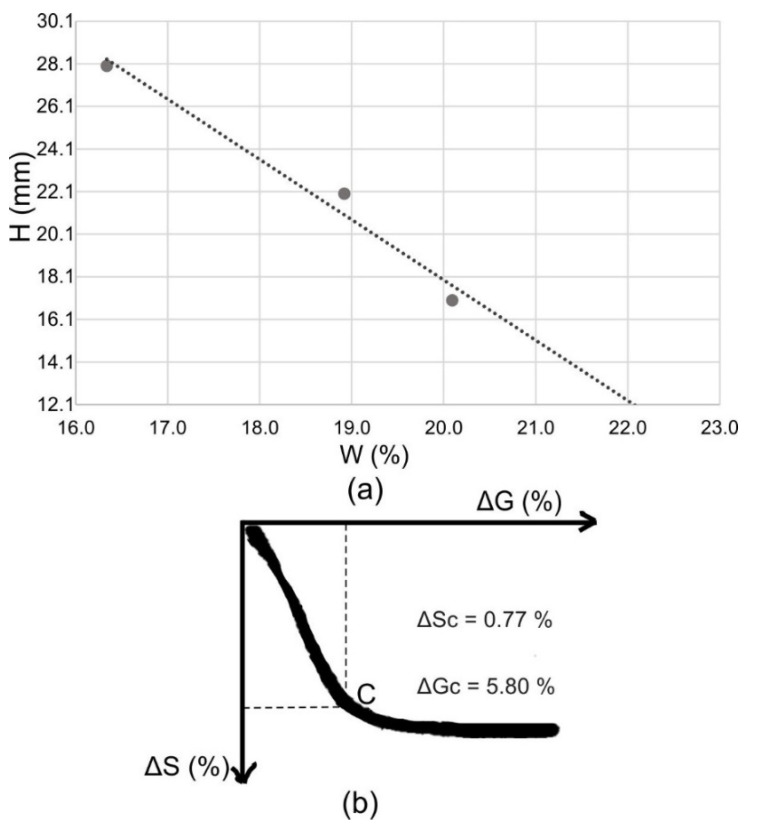
Plasticity according to Pfefferkorn (**a**) and drying susceptibility (Bigot’s curve) (**b**).

Plasticity according to Pfefferkorn and sensitivity to drying were not possible to perform in the aplitic granite waste because the samples were not stable enough for testing, given that they were low in clay minerals (non-plastic material). The coefficient of plasticity of the CGW was 22.1, which classifies the material as moderately plastic (Figure 7a). Water needed for plastic forming of the CGW was determined for a deformation ratio of 2.5 and amounted to 20.7%. The sample was weakly sensitive to drying (Figure 7b), thus experiencing a moist loss during drying in the air of 5.80%, while shrinking to 0.77%.

The important characteristics of the dried and fired samples are shown in Table 4 and Table 5. Low water absorption and modulus of rupture of up to 18.6 MPa after firing of the GW at high temperatures are characteristic of granitic materials [22,27]. The obtained values of water absorption (1.3%) and modulus of rupture (28.8 MPa) in the CGW were similar to the granite sawing waste in ceramic tile formulation from the literature [27]. The bulk density of the composite samples was higher than in the GW, meaning the more intensive consolidation of the matrix appeared during the firing process due to the fluxing action on clay minerals.

**Figure 8:**
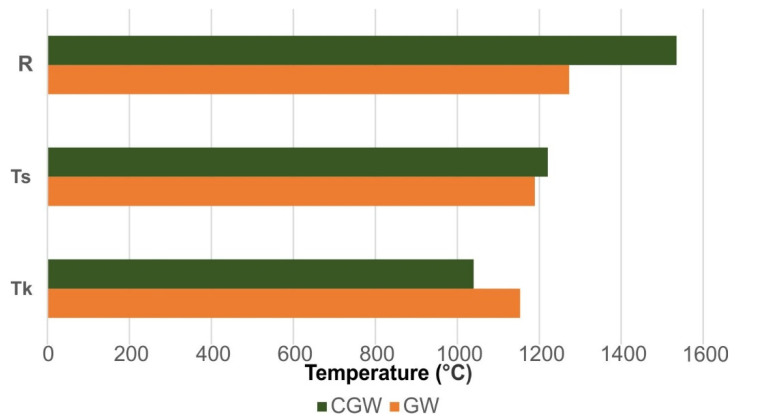
The properties related to firing and softening of the samples (R, refractoriness temperature; T_s_, temperature of sintering; T_k_, temperature of clinkering).

The temperatures of clinkering and sintering, as obtained from the gresification diagram [28], are presented in Figure 8. A very narrow sintering interval of 36 °C in the GW defined by these temperatures range is consistent with the fluxing nature of the test sample. The characteristic temperatures in the case of CGW revealed somewhat lower clinkering and later sintering compared to the GW. The refractoriness of the CGW was obtained much higher (1535 °C) than that of the GW (1273 °C) due to the somewhat higher content of kaolinite and lower amount of quartz. The refractoriness of the sample (GW), presented below, was higher up to about 100 °C than is documented in the literature data for granitic rocks [22].

**Figure 9:**
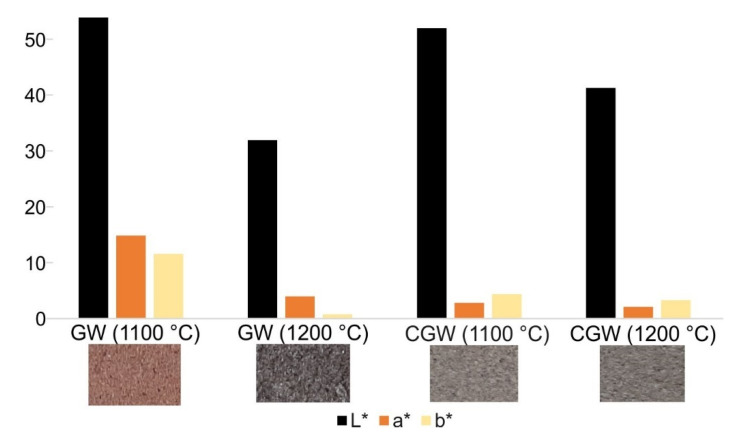
L*a*b* coordinates and the appearance of the surface of the samples.

The appearance of the samples, along with the L*a*b* color coordinates, are shown in Figure 9. The samples of GW fired at 1200 °C seemed overfired due to the appearance of a glassy phase on the sample surface. A grey pattern of satisfying color tonality was obtained in the CGW. Both samples showed an increase in lightness and a decrease in red hues with the firing temperature.

Based on the obtained test results concerning the sample of disintegrated granite, the sample presented young sediment, without plasticity and of insufficient quality in terms of ceramic tile production. Its application is possible as one of the components of the raw material mixture for the production of ceramic tiles.

**Figure 10:**
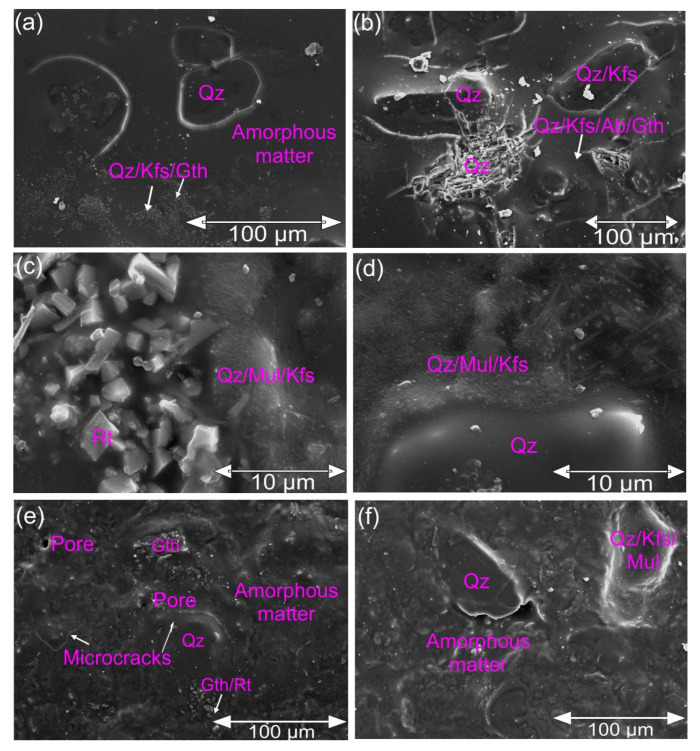
SEM images of the samples fired at 1250 °C: (**a**,**b**) aplitic granite waste; (**c**–**f**) the CGW composite (Qz, quartz; Or, orthoclase; Gth, goethite; Ab, albite; Mul, mullite; Rt, rutile).

SEM-EDS analysis was done on the GW and CGW fired at 1250 °C (Figure 10). The outer side of the samples is recorded below.

The results proved the generally expected mineralogical composition similar to the previous studies [2,40]. Primary mullite was found in the form of nano- and micro-sized elongated crystals in a mixture with feldspars and quartz, as seen previously [2,43]. These elongated crystals are found typically in the case of mullite formed after firing and decomposition of feldspars [20]. Quartz is seen as partly dissolved in the matrix or the form of irregularly shaped crystals [2]. Additionally, the CGW is seen to contain some microcracks, which were mainly around quartz grains due to the α → β conversion [2]. Generally, a usual, porcelain-like, glassy, dense microstructure interrupted by coarser grains of quartz and nano and microcrystals of mullite, feldspars, and quartz is recorded [40]. In addition, a small amount of rounded open pores is noticed in the case of CGW, since the densification of the matrix during melting and sintering is intensified by the addition of the aplitic granite waste [2]. The pores of 2.6–5.2 µm in size are seen in Figure 10, which are significantly smaller than those obtained previously with mineralogically similar waste material [2]. The GW sample contained somewhat more cracks than the CGW, with highly compact parts due to the more liquid phase that formed in the pure GW. Unusual accumulations in the matrix of the CGW presented minor amounts of rutile and goethite.

### 3.3. The Characteristics of the Semi-Industrial Products

The tiles samples of 50 × 120 mm^2^ in size served as a semi-industrial probe and were tested as unglazed tiles according to SRPS EN ISO 10545 group of standards (Table 6). The aesthetic appearance of the products is only rarely given a focus in the literature [44].

The tested tiles were resistant to chemicals, deep abrasion, and frost. According to the water absorption and modulus of rupture they are classified as the SRPS EN 14411—Annex H group (0.5% < Eb ≤ 3%) [50]. The tested tiles can be used for cladding the exterior and interior surfaces of walls and floors. The lead and cadmium contents were below the detection limits.

## 4. Conclusions

The tested aplitic granite waste is a useful alternative raw material for the production of ceramic tiles if mixed with ceramic clays relatively rich in clay minerals. This study shows that the usage of this waste material can contribute to the preservation of the environment by avoiding the need for the disposal and conservation of scarce natural feldspar deposits by way of resource substitution. The obtained ceramic tiles are of good quality, as confirmed by the semi-industrial probe tests according to EN standards. The main conclusions from the study are as follows:

1. The aplitic granite waste contains mainly feldspar (especially albite) and quartz and small amounts of micas and minor kaolinite. As such, the material is suitable as a filler and flux in ceramic batches by introducing feldspars and quartz. Since it lowers the plasticity of clay, it can be mixed with suitable raw materials of a decent quantity of clay minerals;
2. Thermal analysis showed that the pure aplitic granite expands by raising the temperature, and significantly shrinks at the end of testing. This effect is mitigated by the addition of clay;
3. The composite containing 40 mass% of the waste was of moderate plasticity and not susceptible to drying. A firing shrinkage of 2.2% was obtained;
4. The samples fired both at 1200 and 1250 °C satisfied the requirements of the European standard concerning water absorption and modulus of rupture;
5. The waste material is considered safe in terms of leaching of the trace elements.
6. The composite material is observed to contain large quartz grains and a dense matrix interspersed with elongated crystals of mullite.
7. The tiles are proven as freeze/thaw-resistant and not harmful to the environment in terms of lead and cadmium discharges;
8. The semi-industrial probe turns out to meet all the requirements of the standards for unglazed tiles;
9. The lowest obtained lightness of the tiles was found after firing at 1200 °C, and was lowered to about 41 when mixed with clay.

## Figures and Tables

**Table 1:** Chemical composition and particle size distribution of the aplitic granite waste (GW) and the composite (CGW).

Parameter (mass%)	GW	CGW
LOI ^1^	1.10 ± 0.20	3.52 ± 0.30
SiO_2_	71.76 ± 0.50	65.96 ± 0.40
Al_2_O_3_	14.42 ± 0.40	20.59 ± 0.45
TiO_2_	0.21 ± 0.08	0.61 ± 0.10
Fe_2_O_3_	1.54 ± 0.25	1.36 ± 0.30
CaO	1.36 ± 0.20	0.52 ± 0.10
MgO	0.76 ± 0.20	1.36 ± 0.25
Na_2_O	3.57 ± 0.20	2.18 ± 0.10
K_2_O	4.65 ± 0.25	3.60 ± 0.30
SO_3_	0.02 ± 0.01	0.02 ± 0.01
P_2_O_5_	0.13 ± 0.02	0.25 ± 0.05
MnO	0.06 ± 0.02	0.02 ± 0.01
Total carbonates contents	0.00	0.00
Clay < 0.002 mm	3 ± 0.6	16 ± 0.6
Alevrolite 0.002 mm < particles < 0.06 mm	37 ± 0.6	54 ± 0.6
Sand > 0.06 mm	60 ± 0.6	30 ± 0.0
Remains on the 0.063 mm sieve	56.37 ± 0.58	31.86 ± 0.58

^1^ LOI—loss on ignition.

**Table 2:** Chemical content of the trace elements in the aplitic granite waste (GW) and the composite (CGW).

Element (mg/kg)	GW	CGW
Pb	10.2 ± 0.1	18.7 ± 0.2
Cd	<0.2	<0.2
Hg	<0.2	<0.2
Cr	35.8 ± 0.1	29.2 ± 0.0
Cu	4.5 ± 0.1	3.7 ± 0.1
Zn	40.0 ± 0.2	39.0 ± 0.2
Ba	220 ± 0.4	167 ± 0.3
Ni	6.4 ± 0.2	8.4 ± 0.2
As	<0.3	<0.3
Re	<0.2	<0.2

**Table 3:** Mineralogical composition of the aplitic granite waste (GW) and the composite (CGW).

Phase ^1^ (mass%)	GW (mass%)	CGW (mass%)
Albite (Ab)	38.5	15.4
Orthoclase (Or)	23.3	11.6
Quartz (Qz)	23.2	47.8
Illite-mica (Ilt-mca)	10.3	19.6
Kaolinite (Kln)	2.0	4.4
Dolomite (Dol)	1.2	0.5
Vermiculite (Ver)	0.6	0.3
Goethite (Gth)	0.9	0.4

^1^ IMA-CNMNC approved symbols are shown in brackets as listed in [33].

**Table 4:** Properties of the aplitic granite waste (GW) tiles on drying and firing.

	Dry Samples			Fired Samples				
Tile Size (mm^2^)	Drying Shrinkage (%)	Modulus of Rupture (MPa)	Firing Temp. (°C)	Firing Shrinkage (%)	Bulk Density (g/cm^3^)	Loss on Ignition (%)	Water Absorption (%)	Modulus of Rupture (MPa)
25 × 120	0.00 ± 0.01	0.75 ± 0.06	1100	0.58 ± 0.05	1.87 ± 0.12	0.85 ± 0.09	12.01 ± 0.11	11.93 ± 0.15
			1200	6.26 ± 0.08	2.10 ± 0.15	0.92 ± 0.09	0.74 ± 0.09	18.02 ± 0.18
50 × 120	0.02 ± 0.02	1.15 ± 0.07	1100	0.42 ± 0.06	1.87 ± 0.11	0.86 ± 0.09	14.85 ± 0.12	11.46 ± 0.14
			1200	6.06 ± 0.08	2.08 ± 0.13	0.87 ± 0.08	0.45 ± 0.06	18.56 ± 0.18

**Table 5:** Properties of the composite mixture (CGW) tiles on drying and firing.

	Dry Samples			Fired Samples				
Tile Size (mm^2^)	Drying Shrinkage (%)	Modulus of Rupture (MPa)	Firing Temp. (°C)	Firing Shrinkage (%)	Bulk Density (g/cm^3^)	Loss on Ignition (%)	Water Absorption (%)	Modulus of Rupture (MPa)
25 × 120	−0.63 ± 0.03	1.13 ± 0.05	1200	3.72 ± 0.07	2.27 ± 0.18	3.56 ± 0.07	2.42 ± 0.08	28.15 ± 0.11
			1250	2.22 ± 0.06	2.06 ± 0.09	3.77 ± 0.07	1.30 ± 0.09	28.85 ± 0.10
50 × 120	−0.54 ± 0.03	1.17 ± 0.04	1200	3.56 ± 0.05	2.23 ± 0.17	3.49 ± 0.06	2.50 ± 0.09	28.24 ± 0.09
			1250	2.24 ± 0.06	2.17 ± 0.15	3.87 ± 0.06	1.33 ± 0.10	28.68 ± 0.09

**Table 6:** The quality of semi-industrial probe from CGW according to SRPS EN ISO 10545.

Property Tested	Sample Firing Temperature	Average Results
Dimensions [45]	1200 °C	48.53 × 116.19 mm^2^
	1250 °C	48.90 × 116.84 mm^2^
Thickness [45]	1200 °C	6.9 mm
	1250 °C	7.0 mm
Surface quality [45]	1200 °C	100% of tiles without defects
	1250 °C	100% of tiles without defects
Water absorption [33]	1200 °C	2.46%
	1250 °C	1.40%
Bending strength [34]	1200 °C	804.4 N
	1250 °C	798.7 N
Modulus of rupture [34]	1200 °C	28.24 MPa
	1250 °C	28.33 MPa
Deep abrasion [46]	1200 °C	225 mm^3^
	1250 °C	225 mm^3^
Linear thermal expansion [47]	1200 °C	0.390 mm/m
	1250 °C	0.380 mm/m
Freeze/thaw resistance [48]	1200 °C	E_1_ = 2.27%, E_1_ = 2.39%; no defects
	1250 °C	E_1_ = 1.29%, E_1_ = 1.32%; no defects
Chemical resistance [49]	1200 °C	Class A
	1250 °C	Class A
Pb and Cd [38]	1200 °C	<0.03 mg/L and <0.01 mg/L
	1250 °C	<0.03 mg/L and <0.01 mg/L

## Data Availability

The data is contained within the article. Additional data are available 533 on request from the corresponding author.

## References

[1] (2022). Eurostat Statistics Explained, Material Flow Accounts and Resource Productivity. https://ec.europa.eu/eurostat/statistics-explained/index.php?title=Material_flow_accounts_and_resource_productivity#Consumption_by_material_category.

[2] Öztürk Ç., Akpınar S., Tarhan M. (2021). Investigation of the usability of Sille stone as additive in floor tiles. J. Aust. Ceram. Soc..

[3] Dondi M. (2018). Feldspathic fluxes for ceramics: Sources, production trends and technological value. Resour. Conserv. Recycl..

[4] Dondi M., García-Ten J., Rambaldi E., Zanelli C., Vicent-Cabedo M. (2021). Resource efficiency versus market trends in the ceramic tile industry: Effect on the supply chain in Italy and Spain. Resour. Conserv. Recycl..

[5] Yilmaz M., Tuğrul A. Usability of granitic rock wastes as asphalt aggregate. Proceedings of the International Conference Sustainable Aggregates Resource Management.

[6] Shilar F.A., Quadri S.S. (2019). Performance evaluation of interlocking bricks using granite waste powder. Int. J. Eng. Appl. Sci. Technol..

[7] Dino G.A., Clemente P., Lasagna M., De Luca D.A. (2013). Residual sludge from dimension stones: Characterisation for their exploitation in civil and environmental applications. Energy Procedia.

[8] Ahmed H.M., Abdelhaffez G.S., Ahmed A.A. (2020). Potential use of marble and granite solid wastes as environmentally friendly coarse particulate in civil constructions. Int. J. Environ. Sci. Technol..

[9] Turkmen O., Kucuk A., Akpinar S. (2015). Effect of wollastonite addition on sintering of hard porcelain. Ceram. Int..

[10] Ostrowski K., Stefaniuk D., Sadowski Ł., Krzywiński K., Gicala M., Różańska M. (2020). Potential use of granite waste sourced from rock processing for the application as coarse aggregate in high-performance self-compacting concrete. Constr. Build. Mater..

[11] Pinter Junior J., Zaccaron A., Arcaro S., Neto J.B.R., de Noni Junior A., Pereira F.R. (2022). Novel approach to ensure the dimensional stability of large-format enameled porcelain stoneware tiles through water absorption control. Open Ceram..

[12] Luo Y., Zheng S., Ma S., Liu C., Wang X. (2017). Ceramic tiles derived from coal fly ash: Preparation and mechanical characterization. Ceram. Int..

[13] Luo Y., Wu Y., Ma S., Zheng S., Chu P.K. (2019). An eco-friendly and cleaner process for preparing architectural ceramics from coal fly ash: Pre-activation of coal fly ash by a mechanochemical method. J. Clean. Prod..

[14] Dondi M., Guarini G., Conte S., Molinari C., Soldati R., Zanelli C. (2019). Deposits, composition and technological behavior of fluxes for ceramic tiles. Period. Di Mineral..

[15] Torres P., Manjate R.S., Quaresma S., Fernandes H.R., Ferreira J.M.F. (2007). Development of ceramic floor tile compositions based on quartzite and granite sludges. J. Eur. Ceram. Soc..

[16] Souza A.J., Pinheiro B.C.A., Holanda J.N.F. (2010). Processing of floor tiles bearing ornamental rock-cutting waste. J. Mater. Process. Technol..

[17] Gadioli M.C.B., de Aguiar M.C., de Andrade Pazeto M., Monteiro S.N. (2012). Influence of the granite waste into a clayey ceramic body for rustic wall tiles. Mater. Sci. Forum.

[18] Araújo A.J.M., Sousa A.R.O., Macedo D.A., Dutra R.P.S., Campos L.F.A. (2019). Effects of granite waste addition on the technological properties of industrial silicate based-ceramics. Mater. Res. Express.

[19] Ngayakamo B., Bello A., Onwualu A.P. (2020). Development of eco-friendly fired clay bricks incorporated with granite and eggshell wastes. Environ. Chall..

[20] Torres P., Fernandes H.R., Olhero S., Ferreira J.M.F. (2009). Incorporation of wastes from granite rock cutting and polishing industries to produce roof tiles. J. Eur. Ceram. Soc..

[21] El-Maghraby A., ElMaaty M.A.A., Khater G.A., Mostafa N.Y. (2010). Utilization of grantitoid rocks in Taif area as raw materials in ceramic bodies. J. Am. Sci..

[22] Poznyak A.I., Levitskii I.A., Barantseva S.E. (2012). Basaltic and granitic rocks as components of ceramic mixes for interior wall tiles. Glass Ceram..

[23] Gaied M.E., Gallala W., Essefi E., Montacer M. (2011). Microstructural and mechanical properties in traditional ceramics as a function of quartzofeldspathic sand incorporation. Trans. Indian Ceram. Soc..

[24] Vrbický T., Přikryl R. (2021). Recovery of some critical raw materials from processing waste of feldspar ore related to hydrothermally altered granite: Laboratory-scale beneficiation. Minerals.

[25] Dino G.A., Fornaro M., Trentin A. (2012). Quarry waste: Chances of a possible economic and environmental valorisation of the Montorfano and Baveno granite disposal sites. J. Geol. Res..

[26] Simić V., Životić D., Miladinović Z. (2021). Towards better valorisation of industrial minerals and rocks in Serbia—Case study of industrial clays. Resources.

[27] Menezes R.R., Navarro L., Santana L., Neves G.A., Ferreira H.C., Srivastava J. (2012). Recycling of Mine Wastes as Ceramic Raw Materials: An Alternative to Avoid Environmental Contamination. Environmental Contamination.

[28] Vasić M.V., Pezo L., Vasić M.R., Mijatović N., Mitrić M., Radojević Z. (2020). What is the most relevant method for water absorption determination in ceramic tiles produced by illitic-kaolinitic clays? The mystery behind the gresification diagram. Bol. Soc. Esp. Cerám. V..

[29] Arsenović M., Pezo L., Mančić L., Radojević Z. (2014). Thermal and mineralogical characterization of loess heavy clays for potential use in brick industry. Thermochim. Acta.

[30] Hillier S. (2000). Accurate quantitative analysis of clay and other minerals in sandstones by XRD: Comparison of a Rietveld and reference intensity ratio (RIR) method and the importance of sample preparation. Clay Miner..

[31] Vasić M.V., Terzić A., Radovanović Ž., Radojević Z., Warr L.N. (2022). Alkali-activated geopolymerization of a low illitic raw clay and waste brick mixture. An alternative to traditional ceramics. Appl. Clay Sci..

[32] Vasić M.V., Goel G., Vasić M., Radojević Z. (2021). Recycling of waste coal dust for the energy-efficient fabrication of bricks: A laboratory to industrial-scale study. Environ. Technol. Innov..

[33] (2018). Ceramic Tiles—Part 3: Determination of Water Absorption, Apparent Porosity, Apparent Relative Density and Bulk Density.

[34] (2019). Ceramic Tiles—Part 4: Determination of Modulus of Rupture and Breaking Strength.

[35] (1997). Methods of Test for Dense Shaped Refractory Products—Part 12: Determination of Pyrometric Cone Equivalent (Refractoriness).

[36] Tossavainen M. (2000). Leaching Behavior of Rock Materials and a Comparison with Slag Used in Road Construction. Ph.D. Thesis.

[37] Haiying Z., Youcai Z., Jingyu Q. (2007). Study on use of MSWI fly ash in ceramic tile. J. Haz. Mat..

[38] (2021). Ceramic Tiles—Part 15: Determination of Lead and Cadmium Given Off by Tiles.

[39] Kurešević L. (2013). Tertiary plutonic rocks of central and western Serbia Vardar zone as dimension stone. Acta Montan. Slovaca Ročník.

[40] Warr L.N. (2021). IMA–CNMNC approved mineral symbols. Miner. Mag..

[41] Hlavay J., Jonas K., Elek S., Inczedy J. (1978). Characterization of the particle size and the crystallinity of certain minerals by IR spectrophotometry and other instrumental methods-II. Investigations on quartz and feldspar. Clay. Clay Miner..

[42] Passaretti M.G., Ninago M.D., Paulo C.I., Petit H.A., Irassarc E.F., Vega D.A., Villar M.A., López O.V. (2019). Biocomposites Based on Thermoplastic Starch and Granite Sand Quarry Waste.

[43] Theodosoglou E., Koroneos A., Soldatos T., Zorba T., Paraskevopoulos K.M. (2010). Comparative Fourier transform infrared and X-ray powder diffraction analysis of naturally occurred K-feldspars. Bull. Geol. Soc. G..

[44] Zanelli C., Conte S., Molinari C., Soldati R., Dondi M. (2021). Waste recycling in ceramic tiles: A technological outlook. Resour. Conserv. Recycl..

[45] (2019). Ceramic Tiles—Part 2: Determination of Dimensions and Surface Quality.

[46] (2015). Ceramic Tiles—Part 6: Determination of Resistance to Deep Abrasion for Unglazed Tiles.

[47] (2015). Ceramic Tiles—Part 8: Determination of Linear Thermal Expansion.

[48] (2012). Ceramic Tiles—Part 12: Determination of Frost Resistance.

[49] (2017). Ceramic Tiles—Part 13: Determination of Chemical Resistance.

[50] (2017). Ceramic Tiles—Definition, Classification, Characteristics Assessment and Verification of Constancy of Performance and Marking.

